# Iron status in women of reproductive age in Switzerland: the role of inflammation and ferritin thresholds for the prevalence of iron deficiency–a cross-sectional study

**DOI:** 10.1038/s41430-025-01685-z

**Published:** 2025-11-28

**Authors:** Isabelle Herter-Aeberli, Maria Andersson, Valeria Galetti

**Affiliations:** 1 Laboratory of Nutrition and Metabolic Epigenetics, Institute of Food, Nutrition, and Health, ETH Zurich, Zurich, Switzerland; 2 Nutrition Research Unit, Children’s Research Centre, University Children’s Hospital Zurich, Zurich, Switzerland; 3 GroundWork, Fläsch, Switzerland

**Keywords:** Biomarkers, Diseases

## Abstract

**Background/Objectives:**

Iron deficiency in women of childbearing age remains a public health challenge, but prevalence data in high-income countries is scarce and the role of predictors remains uncertain. We determined the prevalence of iron deficiency in women in Switzerland and assessed the influence of BMI, inflammation, and age on iron status. In addition, we determined the ferritin concentration below which hemoglobin (Hb) starts to decline.

**Subjects/Methods:**

This is a secondary, pooled data analysis including data from 26 studies conducted in Switzerland between 2009 and 2020. Participants were a convenience sample of generally healthy women aged between 18 and 54 years (*n* = 2709).

**Results:**

The prevalence of iron deficiency in women (median 23.3 years; IQR: 21.1–26.4) was 18.9%, while 4.7% of the women were anemic and 3.3% were iron deficient anemic. The prevalence of overweight (BMI ≥ 25 kg/m^2^) was 7.2%, and 1.4% were obese (BMI ≥ 30 kg/m^2^); 8.9% suffered from acute inflammation (CRP ≥ 5 mg/l). In multivariate regression analysis, BMI and age were positive predictors of ferritin (*p* < 0.001), while inflammation was not. Correcting iron status for inflammation had a negligible effect on the prevalence of iron deficiency. We observed a decrease in Hb below a ferritin concentration of 28.5 µg/l.

**Conclusions:**

In this convenience sample of young women in Switzerland, one in five was iron deficient and one in 30 was anemic due to iron deficiency. Controlling ferritin concentrations for inflammation did not substantially affect the prevalence of iron deficiency, indicating that such corrections are redundant in a healthy population with a low prevalence of inflammation. Impaired erythropoiesis was observed when the ferritin concentration fell below 28.5 µg/l, providing further evidence for a physiologically based ferritin threshold to identify the onset of iron-deficient erythropoiesis.

## Introduction

The global prevalence of anemia among women of reproductive age is 30% [1], and iron deficiency is estimated to account for 50% of the cases, though the prevalence varies by region [2]. Women of reproductive age are at risk for iron deficiency as a result of low dietary iron intakes, poor iron bioavailability, and iron losses during menses, or a combination of all [3, 4]. Studies in Europe report prevalence rates of iron deficiency ranging from 3.1 to 32%, but few studies are nationally representative [5, 6].

A large body of epidemiological evidence demonstrates an increased risk for iron deficiency in overweight or obese women [7–12]. Obesity is a state of low-grade systemic inflammation which is reflected in an array of elevated pro-inflammatory cytokines and adipokines [13]. Hepcidin, the main iron regulatory protein in the human body, binds to the cellular iron export protein ferroportin, leading to its internalization, degradation, and reduced release of iron into the circulation [14]. At the transcriptional level, hepcidin is regulated through different pathways. The primary regulation is based on body iron stores. High body iron stores upregulate hepcidin through the BMP/SMAD pathway and reduce iron release from the enterocytes, macrophages, and hepatocytes. Pro-inflammatory cytokines, namely interleukin 6, upregulate hepcidin production through the JAK-STAT pathway with a similar effect [14, 15]. It has been suggested that the reduced iron status observed in obesity may be due to a reduction in iron absorption [16–18], but not all studies agree [19, 20].

Serum or plasma ferritin is the recommended and most commonly used marker for the determination of iron status in population studies [21]. In clinical practice, serum ferritin and plasma ferritin are often used interchangeably, as both refer to the same protein. For the sake of simplicity, throughout this article, we will use the term “ferritin” to refer to both serum and plasma ferritin. Traditionally, based on expert opinion, a ferritin concentration <15 µg/l is used to define iron deficiency [21]. Ferritin concentrations in serum or plasma reflect the circulating fraction of the body’s ferritin pool and are reflective of iron stores [22]. However, accumulating evidence now suggests that this cut-off may be set at a higher level, since the body starts adapting its iron physiology well before reaching this concentration [4]. In addition, the interpretation of ferritin is difficult, especially in the context of inflammation. Ferritin, being an acute phase protein, increases in response to inflammation independent of iron stores. Different methods to correct ferritin concentrations for inflammation are available [21, 23, 24]. However, to date, these correction methods were primarily evaluated, applied, and found useful in settings with high rates of inflammation [25, 26]. Adjustment using the BRINDA method is generally recommended only when a statistical association between the biomarker and inflammatory markers is observed [27]. The usefulness of correction methods for the interpretation of ferritin concentrations in high-income settings with low prevalence of inflammation is unclear.

Data on iron intake and iron status in women of reproductive age in Switzerland is scarce. The first national nutrition survey conducted in 2014–2015 assessed iron intake in the Swiss population using two 24-h recalls per participant [28]. This study suggests that women of reproductive age consume only 60% of the recommended daily iron intake of 15 mg [29]. Based on these findings, it can be expected that a significant proportion of women may develop iron deficiency. However, no biomarkers of iron status were measured in this study. A recent study investigated the incidence of iron deficiency with or without anemia in the Swiss primary care system using different ferritin cut-offs. At a cut-off of 15 µg/l, the incidence was 10.9 cases per 1000 patient-years, comprising both males and females, while it increased to 29.9 cases per 1000 patient years with a cut-off of 30 µg/l [30].

The objectives of this study in young women in Switzerland were: to (1) determine the prevalence of iron deficiency; (2) identify factors influencing iron status; (3) determine the ferritin threshold below which hemoglobin starts decreasing; and (4) assess the effect of different correction methods for inflammation on the prevalence of iron deficiency.

## Subjects and methods

### Study design

In this secondary analysis, we included data from 26 studies conducted in Switzerland between 2009 and 2020 (Supplementary Table 1). Detailed descriptions of the individual studies can be found elsewhere [16, 31–46]. Out of the 26 studies, 24 studies investigated iron absorption in young women from various test meals and/or iron compounds using stable isotopes. One study [36] was an intervention trial of iron fortification. For the current analysis, we used screening data from these 25 studies. For the screenings, healthy women in the relevant age range were invited and screened for weight, iron status, and inflammation. The last study was a national sample of women of reproductive age [35]. The primary outcome was ferritin concentration, while C-reactive protein (CRP) and hemoglobin (Hb) were secondary outcomes, and BMI and age potential confounders. This analysis, and all the original studies, were conducted ensuring adherence to good clinical practice, the guidelines laid down in the Declaration of Helsinki, as well as applicable regulatory requirements. The studies’ procedures were approved by the Cantonal Ethical Committee of Zurich. The current analysis was approved by the Cantonal Ethical Committee of Zurich under the number BASEC Nr. 2020-02399. Written informed consent was obtained from all participants in the original studies.

### Participants

Participants in the absorption studies and the intervention study were predominantly recruited among the student and staff population of ETH Zurich and the University of Zurich, Switzerland, through e-mail lists and flyers. Participants in the national survey were recruited through nine obstetric/prenatal care clinics/hospitals throughout Switzerland and were invited to participate during their routine visits [35]. We included generally healthy (no report of metabolic, gastrointestinal, kidney or other chronic disease), non-pregnant and non-lactating, non-smoking women aged between 18 and 54 years (for most studies 18–45 years). Significant blood loss or blood donation within the past 4 months was an exclusion criterion for all iron absorption studies. Although a large proportion of the iron absorption studies had specific inclusion criteria for body weight or BMI, including participants with a body weight <65 or 70 kg and a body mass index (BMI) < 25 kg/m^2^, women with higher weight and/or BMI were screened. For the intervention study [36] women with a BMI > 28.5 kg/m^2^ were excluded, but were included in the screening. In this secondary data analysis, we included participants with at least a valid ferritin and CRP measurement and with consent for re-use of data.

### Laboratory analyses

All laboratory analyses were performed at the time of the original studies. We measured Hb in venous whole blood on the day of venipuncture by using a Coulter Counter (Beckman Coulter, Life Sciences, Indianapolis, USA), a Sysmex Analyser (XE_5000, Sysmex Europe GmbH, Norderstedt, Germany), or an ABX Pentra 60 C+ hematology analyzer (Horiba group, France). We separated serum or plasma by centrifugation and stored aliquots at -20° C until analysis. We measured serum or plasma ferritin by using a chemiluminescent immunoassay on an Immulite automatic system (Siemens Healthcare GmbH, Erlangen, Germany), including certified controls for each run, or a multiplex sandwich ELISA at VitMin Laboratory [47]. We measured CRP by using a chemiluminescent immunoassay on an Immulite automatic system (Siemens Healthcare GmbH, Erlangen, Germany), or on a multiplex sandwich ELISA at VitMin Laboratory [47]. We pooled data from these two assays because the comparison between the VitMin Laboratory ELISA assays and different standard assays (including Roche Cobas as well as different commercial RIA and ELISA assays) for ferritin and CRP has shown comparable results [47, 48]. Assay-specific external quality control samples were used for all analyses.

### Statistical analysis

We analyzed data using IBM SPSS statistics (version 26) and R statistical programming environment (version 4.3.3, R Core Team 2020, R Foundation for Statistical Computing, Vienna, Austria).

We calculated BMI from measured weight and height as follows: BMI (kg/m^2^) = weight (kg)/height^2^ (m). We defined overweight as a BMI ≥ 25 kg/m^2^ and obesity as a BMI ≥ 30 kg/m^2^. We defined the presence of inflammation as a CRP ≥ 5 mg/l, iron deficiency as a ferritin <15 µg/l, anemia as an Hb <12 g/dl, and iron deficiency anemia as a ferritin < 15 µg/l combined with an Hb <12 g/dl [21, 49, 50]. Clinical guidelines also use a ferritin cut-off of 30 µg/l, thus we calculated iron deficiency based on this as well [51].

We checked all data for normality visually as well by using the Kolmogorov-Smirnov test. We present normally distributed data using mean ± standard deviation and non-normally distributed data using median (min-max). We assessed correlations between ferritin, Hb, BMI, CRP, and age by using Spearman’s rank correlation analysis. We present the results of the correlations with the Spearman’s rank correlation rho (r) and significance level (p). We used linear multivariate regression analysis to determine predictors of ferritin and Hb concentration. The first model included ferritin as the dependent variable and BMI, age, and CRP as independent variables. As CRP was not a significant predictor in this model, we ran a second model excluding CRP. The third model included Hb as the dependent variable and ferritin, BMI, age, and CRP as independent variables. As BMI was not a significant predictor in this model, we ran a fourth model excluding BMI. As the residuals were not normally distributed using untransformed values in the first and second models, we used log-transformed ferritin values for the regression analysis in these two models. We present the results of the linear multivariate regressions with the coefficient (B), the standardized coefficient (Beta), the significance level (p), and the coefficient of determination (R^2^). The significance level was set at p < 0.05.

To visualize the relationship between crude ferritin and Hb concentrations, we applied a generalized additive mixed effect model (GAMM) fit using R packages lme4 (version 1.1-35.2), mgcv (version 1.9-1), and ggplot2 (version 3.5.0), as previously described [52, 53]. We defined “study” as the random factor to account for the clustered structure of the data. We present the GAMM with the significance level (p) for the evaluation of the fitted smoother’s difference from a null (linear) model, the adjusted coefficient of determination (R^2^). We used the method of finite differences to estimate the first derivative (indicative of the instantaneous rate of change) of the fitted GAMM smoother to identify notable concentrations of ferritin below which the association between ferritin and Hb is significant (i.e., visible when the GAMM model’s derivative is significantly different from zero) [52, 53].

We used five different adjustment methods to investigate the impact of CRP on the prevalence of iron deficiency: (1) we excluded all participants with CRP ≥ 5 mg/l for the calculation of the prevalence of iron deficiency [21]; (2) we adjusted the cut-off for iron deficiency in those participants with CRP ≥ 5 mg/l to a ferritin of 30 µg/l [21]; (3) we used the correction factor of 0.77 proposed by Thurnham et al. [24] on the ferritin concentrations of those participants with CRP ≥ 5 mg/l; (4) we calculated an internal correction factor for ferritin concentrations in participants with CRP ≥ 5 mg/l as proposed by Namaste et al. [23] (the internal correction factor was 0.965); and (5) we calculated an internal regression for correcting ferritin concentrations as proposed by Namaste et al. [23, 27]; our regression was the following: ln(ferritin_adjusted_) = ln(ferritin_unadjusted_) – 0.018* (ln(CRP_observed_) + 2.216); we applied this regression in all participants with CRP > 0.109 (internal first decile).

## Results

The 26 studies included in this analysis resulted in 2839 eligible participants. Data of participants who declared non-consent for re-use of their data in the original studies (*n* = 130) was not transferred for pooling. Thus, data from 2709 participants was used for the pooled analysis. Of those, 7 participants had incomplete records for weight, height, and BMI, while 167 had missing age data, and 17 lacked Hb data. Participants with missing values were excluded from the analyses concerned.

The demographic and anthropometric characteristics of the participants including iron and inflammatory status are shown in Table 1. The mean (IQR) age of the participants was 23.3 (21.1–26.4) years with only 5 participants aged 45 years and older. Based on crude data, the prevalence of iron deficiency (ferritin <15 µg/l) was 18.9% (*n* = 513), while 4.7% (*n* = 127) of the women presented with anemia, and 3.3% (*n* = 90) were iron deficient anemic. When using a ferritin cut-off of <30 µg/l to define iron deficiency, the prevalence increased to 45.8% (*n* = 1241), and 4.2% (*n* = 113) were iron deficient anemic. The prevalence of overweight in the study population was 7.2% (*n* = 195) and 1.4% (*n* = 38) were obese. Acute inflammation was found in 8.9% (*n* = 439) of the women.

**Table 1:** Participant characteristics of a pooled dataset from 26 studies conducted in Switzerland between 2009 and 2020.

	*N*	
Age (y)	2542	23.3 (21.1–26.4)^a^
Weight (kg)	2702	60.0 ± 7.78^b^
Height (m)	2702	1.67 ± 0.06
BMI (kg/m^2^)	2702	21.6 ± 2.6
Hb (g/dl)	2692	13.6 ± 1.0
Ferritin (µg/l)	2709	32.6 (18.3–51.7)
CRP (mg/l)	2709	0.66 (0.26–1.87)

*BMI* body mass index, *CRP* C-reactive protein, *Hb* hemoglobin.

^a^Median (IQR) (all such values).

^b^Mean ± SD (all such values).

Spearman correlation analysis revealed positive associations between ferritin and Hb (r = 0.272, *p* < 0.001), ferritin and BMI (r = 0.066, *p* = 0.001), and ferritin and age (r = 0.057, *p* = 0.004), but not between ferritin and CRP (r = 0.030, p = 0.116). Furthermore, BMI was positively associated with CRP (r = 0.206, *p* < 0.001), and with age (r = 0.095, *p* < 0.001). The same correlations were investigated when data from all participants with elevated CRP (as an indicator of acute inflammation) were removed. The results of these analyses were comparable and can be found in Supplementary Table 2.

In a multivariate regression analysis with ferritin as the dependent variable and BMI, CRP, and age as the independent variables, BMI and age remained weak predictors (B = 0.010, SE = 0.003, *p* < 0.001 for BMI and B = 0.005, SE = 0.001, *p* < 0.001 for age), while CRP was not (B = 0.018, SD = 0.025, *p* = 0.460) (model 1 in Table 2). The model did not change when CRP was removed (model 2). In another regression analysis with Hb as the dependent variable and BMI, age, CRP and ferritin as the independent variables, age, CRP and ferritin remained significant predictors (B = –0.014, SE = 0.004, *p* < 0.001 for age, B = –0.177, SE = 0.063, *p* = 0.005 for log CRP and B = 0.973, SE = 0.050, *p* < 0.001 for log ferritin), while BMI did not (B = 0.007, SE = 0.007, *p* = 0.304) (model 3). The model characteristics did not change by removing BMI from the model (model 4). All regression models were also run when data from participants with inflammation were removed. The results of these analyses were comparable (Supplementary Table 3).

**Table 2:** Linear regression analysis for the prediction of ferritin concentrations in 2709 women in Switzerland.

Model	Dependent	Independent	B	SE	Beta	*p*	R^2^	SE
1	Log ferritin	BMI	0.010	0.003	0.075	<0.001	0.014	0.359
		Age	0.005	0.001	0.073	<0.001		
		Log CRP	0.018	0.025	0.015	0.460		
2	Log ferritin	BMI	0.011	0.003	0.078	<0.001	0.013	0.359
		Age	0.005	0.001	0.072	<0.001		
3	Hb	BMI	0.007	0.007	0.020	0.304	0.134	0.905
		Age	–0.014	0.004	–0.071	<0.001		
		Log CRP	–0.177	0.063	–0.053	0.005		
		Log Ferritin	0.973	0.050	0.362	<0.001		
4	Hb	Age	–0.014	0.004	–0.068	0.002	0.133	0.905
		Log CRP	–0.162	0.061	–0.049	0.008		
		Log Ferritin	0.976	0.050	0.363	<0.001		

*B* unstandardized coefficient, *Beta* standardized coefficient, *BMI* body mass index, *CRP* C-reactive protein, *Hb* hemoglobin, *SE* standard error.

**Fig. 1:**
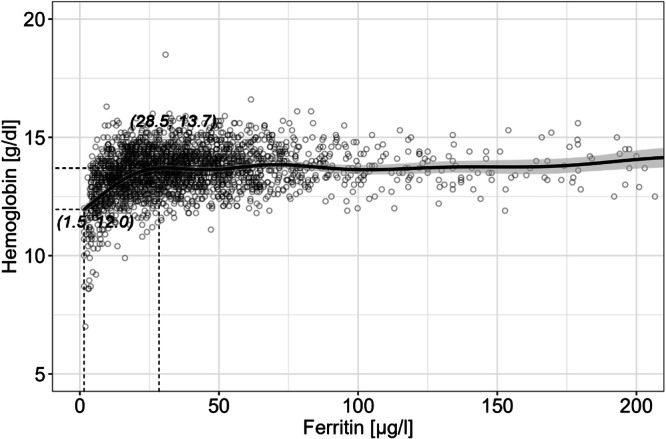
Ferritin versus hemoglobin in healthy adult women (*n* = 2453), excluding inflamed subjects (based on a CRP ≥ 5 mg/l). For greater clarity, observations with a ferritin >210 µg/l are not shown (*n* = 15). The solid line shows the fitted values using GAMM and the shaded areas show the upper and lower 95% confidence intervals around the estimates. The highlighted rectangular areas show the ferritin range where the model’s slope significantly differs from zero, according to the estimation of the model’s first derivative (Supplementary Fig. 1). The derivatives in Supplemental Fig. 1 indicate that the model’s slope is significantly positive from a ferritin value of 1.5 until 28.5 µg/l showing that within this ferritin range hemoglobin increases significantly from 12.0 to 13.7 g/dl (*p* < 0.001, R^2^ = 0.171). When ferritin is greater than 28.5 µg/l, hemoglobin remains stable.

To determine the type of relationship, the associations between ferritin and CRP, ferritin and Hb, ferritin and age, and ferritin and BMI were further investigated using GAMM. A linear relationship was confirmed between ferritin and CRP, between ferritin and age and between ferritin and BMI (data not shown). As expected, we observed a non-linear association between ferritin and Hb (Fig. 1). Using the derivative of the GAMM model, we show that the slope was significantly different from zero for a ferritin value from 1.5 to 28.5 µg/l corresponding to an Hb increase from 12.0 to 13.7 g/dL (*n* = 2453, *p* < 0.001, R^2^ = 0.171). This means that within this range, ferritin was significantly positively associated with Hb (compare supplementary Fig. 1, where the 95% confidence intervals around the derivative does not include zero). The analysis was run excluding all participants with inflammation (*n* = 239). The analysis was repeated by including participants with inflammation and results were comparable (*n* = 2692, *p* < 0.001, R^2^ = 0.161; data not shown).

To determine the effect of inflammation on the prevalence of iron deficiency, we used different correction methods and re-calculated iron deficiency after correction. Depending on the method chosen, the prevalence of iron deficiency in the included women ranged from 18.9% using crude values to 21.3% when using an adjusted ferritin cut-off in participants with inflammation. An overview of the median ferritin concentrations as well as the prevalence of iron deficiency using all the different adjustments is given in Table 3.

**Table 3:** Prevalence of iron deficiency in women of reproductive age in Switzerland using different correction factors accounting for inflammation.

	Unadjusted	Adjustment methods				
		1)	2)	3)	4)	5)
*n*	2709	2467	2709	2709	2709	2709
Ferritin (median (IQR))	32.6 (18.3, 51.7)	32.5 (18.3, 51.6)	32.6 (18.3, 51.7)	31.8 (17.8, 51.1)	32.5 (18.2, 51.5)	31.5 (17.7, 50.1)
Iron deficiency* % (95%CI)	18.9 (17.5, 20.5)	18.9 (17.4, 20.5)	21.3 (19.8, 22.8)	19.6 (18.1, 21.1)	19.0 (17.6, 20.5)	19.6 (18.2, 21.2)

^*^Ferritin < 15 µg/l for all calculations, except for adjusted method 2).

*CRP* C-reactive protein.

1) Participants with CRP ≥ 5 mg/l were excluded from the calculation; 2) the ferritin cut-off for iron deficiency was set at 30 µg/l for those participants with CRP ≥ 5 mg/l; 3) correction using the Thurnham correction factor of 0.77 for those participants with CRP ≥ 5 mg/l [24]; 4) internal correction factor calculated according to Namaste et al. [23] 0.965 for those participants with CRP ≥ 5 mg/l; 5) correction using an internal regression according to BRINDA [23]: lnFER_adj_ = lnFER_unadj_ – 0.018* (lnCRP + 2.216) in those participants with CRP > the internal reference of 0.109 (first decile).

## Discussion

This is the first study providing estimates of the prevalence of iron deficiency in women of childbearing age in Switzerland. Our pooled analysis including data from over 2700 women show that about 1 in 5 young women in Switzerland are iron deficient based on a ferritin cut-off of <15 µg/l, while about 1 in 20 are anemic and 1 in 30 are anemic due to iron deficiency. There is no consensus on the ferritin cut-offs to define iron deficiency and guidelines recommend values ranging from 15 µg/l to 45 µg/l [21, 51]. When raising the ferritin cut-off to 30 µg/l in our sample, almost 1 in 2 women are identified as iron deficient. Adjustment of the cut-off impacts the reported prevalence rate and ultimately also the intervention strategies and treatment decisions [30, 51].

The prevalence of iron deficiency in the young women in our study is consistent with findings from other industrialized countries [5, 6, 12, 54], although the range is large and representative recent data is scarce. While many studies use ferritin alone to determine iron deficiency, the NHANES study 2017–2020 defined absolute and functional iron deficiency using ferritin and transferrin saturation. In women between 18 and 50 years, 34% were shown to have absolute iron deficiency (based on ferritin <30 µg/l only), while 19% had functional iron deficiency (based on ferritin <30 µg/l and transferrin saturation <20%), indicating the impact of the definition of deficiency or parameters used [54]. A recent Swiss study investigated the incidence of iron deficiency and iron deficiency anemia diagnoses in primary health care data using different ferritin cut-offs. They identified 10.9 and 29.9 incident cases per 1000 patient years using a cut-off of 15 and 30 µg/l, respectively [30]. The analysis did not differentiate between male and female patients, but ferritin was more commonly measured in women. Harmonization of the criteria and ferritin cut-offs is needed for uniform definition and diagnosis of iron deficiency.

Ferritin concentration is currently the primary measure for iron deficiency at the population level [21]. Ferritin is responsive to iron interventions and the available laboratory methods are well established [21]. However, current evidence suggests that the body adapts its iron physiology well before ferritin concentration reaches the current cut-off of <15 µg/l for absent bone marrow iron and that the ferritin threshold for iron deficiency may be defined at a higher level, i.e. at the onset of iron-deficient erythropoiesis, before it becomes dysfunctional [4]. Using non-linear modeling of ferritin versus hemoglobin and ferritin versus soluble transferrin receptor, Mei et al. identified a ferritin threshold for non-inflamed women of reproductive age in the US of 25.2 µg/l, above which hemoglobin remains constant and below which hemoglobin starts decreasing and erythropoiesis starts being impaired due to low iron stores [55]. Similarly, Addo et al. identified a threshold of 24.8 µg/l using the same indicators in an international sample for >18,000 women [56]. Using a similar approach but investigating the relationship between iron absorption and ferritin and between iron absorption and hepcidin, Galetti et al. identified a ferritin plateau in non-inflamed women of reproductive age at ≈50 µg/l, above which absorption of dietary iron is constant (at ≈6%) and below which iron absorption starts being gradually upregulated (≈20%) [52]. This suggests that in women of reproductive age, iron regulation adjusts by increasing the rate of dietary iron absorption when ferritin falls below 50 µg/l (i.e., iron stores become depleted) and by limiting iron utilization during erythropoiesis when ferritin falls below 25 µg/l. A ferritin concentration below 15 µg/l, however, is indicative of depleted bone marrow iron stores [57]. We found a strong positive association between ferritin and Hb up to a ferritin concentration of 28.5 µg/l and an Hb of 13.7 g/dl, with no significant association thereafter. This suggests that in our study population reduced iron stores impact erythropoiesis below a ferritin of 28.5 µg/l, which is comparable to the results by Mei et al. [55] and Addo et al. [56]. As expected, our data shows that higher iron stores above this cut-off do not result in higher production of Hb. Although iron absorption is likely still upregulated up to a threshold of 50 µg/l [52], the direct functional benefits between a ferritin of ≈25 µg/l and 50 µg/l are uncertain. In our study, the association between ferritin and Hb was not influenced when excluding inflamed participants, but the prevalence of inflammation was low.

Ferritin is an acute phase protein and its interpretation becomes difficult in populations with high levels of inflammation [22]. Several strategies have been developed to overcome this difficulty, including correction factors [24], regression analysis [23], and altered cut-offs in case of elevated inflammation [21]. However, such strategies were primarily developed for the interpretation of data from populations with high levels of infection, including malaria. For example, in a study in Kenyan pregnant women prevalence of iron deficiency increased by 15-25% when excluding participants with high CRP or applying internal BRINDA regression [26]. Similarly, a study in women of reproductive age in India found an increased prevalence rate of 30% after BRINDA adjustment [25]. In both studies, the inflammatory burden of the participants was high. To determine the usefulness of ferritin correction in a healthy population with low rates of inflammation, we used five different approaches. The prevalence of iron deficiency was 18.9% with uncorrected values and stayed the same when removing inflamed participants from the analysis. However, when increasing the cut-off for iron deficiency to 30 µg/l in participants with inflammation, the overall prevalence of iron deficiency increased to 21.3%. Such a small increase ( + 2.4%) would nevertheless result in a change in the classification of iron deficiency severity in nationally representative studies, where the magnitude of iron deficiency as a public health problem is defined as “mild” for a prevalence of 5.0–19.9% and as “moderate” for a prevalence of 20.0–39.9% [21]. The increase in the prevalence of iron deficiency due to the increase in ferritin threshold, as shown in our study and the primary care study by Jäger et al. [30], may have policy implications. Using the correction factors developed by Thurnham [24] or Namaste [23] or the regression analysis ^(23)^ resulted in a prevalence between 19.0 and 19.6% (but no change in iron deficiency classification). Based on the small differences in prevalence calculated using the different inflammation correction strategies, we conclude that in the setting of a healthy population with relatively low levels of inflammation, i.e., below 10% (in our sample 8.9% were inflamed), a correction of ferritin concentrations may not be necessary primary to the determination of iron deficiency prevalence. Many guidelines recommend including a screening for inflammation and anemia in the work up of iron deficiency [51], but in Switzerland, ferritin testing is accompanied by hemoglobin testing in about 70% of the instances and by CRP testing in less than 50% of the cases [30]. Even though we show correcting for inflammation may not be necessary from an epidemiological point of view, it may still be important for the interpretation and potential treatment at the individual level.

The epidemiological evidence for a negative association between BMI, especially in the obese range, and iron status or a higher prevalence of iron deficiency in women is well established [7–12]. This association is primarily explained by reduced iron absorption in the obese state as a result of chronic, low-grade inflammation [16]. Although BMI was significantly yet weakly positively associated with CRP in our study sample, ferritin was not associated with CRP. In our study population, the prevalence of overweight and obesity was very low (7.2% and 1.4%, respectively), compared to the general Swiss female population (22.8% and 10.2%, respectively) [58]. In contrast, an analysis of NHANES 2017–2020 data showed a higher prevalence of functional iron deficiency in overweight and obese compared to normal weight groups, and of absolute iron deficiency in the group with BMI between 30 and 34.9 kg/m^2^ compared to normal weight [54]. However, the mean BMI in this study was 30 kg/m^2^, while it was 21.6 kg/m^2^ in our sample. This may have masked potential interactions between BMI and iron status in our study sample.

Our study has strengths and limitations. This large data set, although a convenience sample and not nationally representative, provides a unique opportunity to estimate the extent of iron deficiency in women of reproductive age in Switzerland. Nevertheless, the data was limited to the parameters described, while other risk factors and potential confounders were not assessed. A limitation is that participants for the original studies included in this analysis were screened for low iron status, which was communicated in the invitations. Thus, the study population may slightly overrepresent women with low iron status, although knowing their iron status was not a pre-requisite for participation in the screening where iron status was measured. Still, women with a history of diagnosed iron deficiency may have been more likely to present for the screenings. Nevertheless, our results are in line with previous findings from other European countries [5]. Furthermore, overweight and obese participants were underrepresented in our study sample since overweight and obese women were excluded in many of the original iron absorption studies. Similarly, the age range of our participants is rather narrow, since a big part of the volunteers were university students, and we did only include generally healthy participants, thus potentially excluding those with elevated inflammation due to chronic disease. For the analysis, we have combined data from a large number of surveys over a period of >10 years. Even though quality controls were always used for all assays, we cannot completely exclude the possibility of intra-assay changes over time. Furthermore, we combined data from two different assays in this analysis. And even though good agreement was shown in past studies [47, 48], it was not perfect. Lastly, ferritin correction methods suggest including inflammation marker α-1-acid glycoprotein (AGP), which remains high in convalescence as opposed to CRP [23, 24]. This marker is particularly useful in settings where inflammation stems from systemic long-term conditions rather than acute infections. When measured in combination with CRP, it was observed that the magnitude of ferritin change is highest when both CRP and AGP (i.e., > 1 g/L) are elevated. However, in our study sample, AGP levels were not systematically measured.

To conclude, in this large sample of young, healthy women in Switzerland, we found one in five women to be iron deficient and one in 20 to be anemic. Furthermore, we showed that Hb decreased and erythropoiesis was negatively affected below a ferritin concentration of 28.5 µg/l, suggesting that improved iron nutrition may have functional benefits below this threshold in a generally healthy population of young women. The prevalence of iron deficiency did not change markedly when ferritin concentrations were controlled for inflammation, indicating that such corrections may not be necessary for population analysis in healthy populations when the rate of inflammation is lower than 10%. A nationally representative survey is needed to get a more generalizable assessment of iron status in the female Swiss population.

## Supplementary information

Supplementary material

## Data Availability

The dataset analyzed during the current study is available from the corresponding author on reasonable request and upon ethical clearance.
