# Supplementary material for: Iron status in women of reproductive age in Switzerland: the role of inflammation and ferritin thresholds for the prevalence of iron deficiency–a cross-sectional study
Source: Eur J Clin Nutr. 2025 Nov 28;80(2):221–7. doi: 10.1038/s41430-025-01685-z (PMC12929052; doi:10.1038/s41430-025-01685-z)
Supplement: Supplementary file 1 — Supplementary material [file 41430_2025_1685_MOESM1_ESM.docx]

**Supplementary material**

**Supplementary table 1** List of studies included in the pooled data analysis on iron status in women in Switzerland

| **Study** | **Year** | **n** | **Study type^1^** | **Age range (y) (for study inclusion)^2^** | **Weight status (for study inclusion)^2^** | **Title published paper from which screening data was used / Study name if unpublished** | **Ref** |
| --- | --- | --- | --- | --- | --- | --- | --- |
| 1 | 2017/2018 | 74 | 1 | 18-45 | n.a. | Direct assessment of body iron balance in women with and without iron supplementation using a long-term isotope dilution method in Benin and Switzerland (Swiss part) | 1 |
| 2 | 2018 | 77 | 1 | 18-45 | n.a | A heat-stable microparticle platform for oral micronutrient delivery (Human study 2) | 2 |
| 3 | 2016 | 118 | 1 | 18-45 | n.a | A heat-stable microparticle platform for oral micronutrient delivery (Human study 1) | 2 |
| 4 | 2018 | 62 | 1 | 18-40 | Weight <65 kg | Higher Extrusion Temperature Induces Greater Formation of Less Digestible Type V and Retrograded Starch in Iron-Fortified Rice Grains but Does Not Affect Iron Bioavailability: Stable Isotope Studies in Young Women | 3 |
| 5 | 2019 | 148 | 1 | 18-45 | BMI <25 kg/m2 and weight <70 kg | Acute consumption of prebiotic galacto-oligosaccharides increases iron absorption from ferrous fumarate, but not from ferrous sulfate and ferric pyrophosphate: stable iron isotope studies in iron-depleted young women | 4 |
| 6 | 2019 | 125 | 1 | 18-45 | BMI <25 kg/m2 and weight <70 kg | Consumption of galacto-oligosaccharides increases iron absorption from ferrous fumarate: a stable iron isotope study in iron-depleted young women | 5 |
| 7 | 2010 | 672 | 2 | 18-40 | BMI 17-28.5 kg/m2 | Random serial sampling to evaluate efficacy of iron fortification: a randomized controlled trial of margarine fortification with ferric pyrophosphate or sodium iron edetate | 6 |
| 8 | 2013 | 10 | 1 | 18-50 | 18.5-25 kg/m2 and weight <65 kg | Unpublished: Iron absorption from GDS capsules | - |
| 9 | 2012 | 60 | 1 | 18-40 | 18.5-25 kg/m2 and weight <65 kg | Addition of whole wheat flour during injera fermentation degrades phytic acid and triples iron absorption from fortified tef in young women | 7 |
| 10 | 2013 | 64 | 1 | 18-45 | BMI 18.5-39.9 kg/m2 | In overweight and obese women, dietary iron absorption is reduced and the enhancement of iron absorption by ascorbic acid is one-half that in normal-weight women 1 | 8 |
| 11 | 2015 | 128 | 1 | 18-45 | 18.5-26.5 kg/m2 and weight <80 kg | Iron absorption from oral iron supplements given on consecutive versus alternate days and as single morning doses versus twice-daily split dosing in iron-depleted women: two open-label, randomised controlled trials (Part 1) | 9 |
| 12 | 2017 | 73 | 1 | 18-45 | 18.5-26.5 kg/m2 and weight <80 kg | Iron absorption from oral iron supplements given on consecutive versus alternate days and as single morning doses versus twice-daily split dosing in iron-depleted women: two open-label, randomised controlled trials (Part 2) | 9 |
| 13 | 2010 | 71 | 1 | 18-45 | n.a. | Unpublished: The effect of meat components on iron absorption | - |
| 14 | 2009 | 56 | 1 | 18-40 | 19-25 kg/m2 and weight <65 kg | Inulin modifies the bifidobacteria population, fecal lactate concentration, and fecal pH but does not influence iron absorption in women with low iron status | 10 |
| 15-17 | 2009 | 73 | 1 | 18-39 | BMI 18.5 to 25 kg/m2 | Circulating non-transferrin-bound iron after oral administration of supplemental and fortification doses of iron to healthy women: a randomized study | 11 |
| 18 | 2013 | 69 | 1 | 18-40 | 18.5-25 kg/m2 and weight <65 kg | A Higher Proportion of Iron-Rich Leafy Vegetables in a Typical Burkinabe Maize Meal Does Not Increase the Amount of Iron Absorbed in Young Women | 12 |
| 19 | 2014 | 178 | 1 | 18-27 | Weight <66 kg | Sodium pyrophosphate enhances iron bioavailability from bouillon cubes fortified with ferric pyrophosphate | 13 |
| 20 | 2010 | 88 | 1 | 18-40 | 18.5-25 kg/m2 and weight <65 kg | Sodium iron EDTA and ascorbic acid, but not polyphenol oxidase treatment, counteract the strong inhibitory effect of polyphenols from brown sorghum on the absorption of fortification iron in young women | 14 |
| 21 | 2015 | 177 | 3 | 18-44 | n.a. | Effectiveness of increased salt iodine concentration on iodine status: trend analysis of cross-sectional national studies in Switzerland (women of reproductive age) | 15 |
| 22 | 2019 | 61 | 1 | 18-45 | 18.5-25 kg/m2 and weight <70 kg | Kinetics of iron absorption from ferrous fumarate with and without galacto-oligosaccharides determined from stable isotope appearance curves in women. | 16 |
| 23 | 2019 | 124 | 1 | 18-45 | 18.5-25 kg/m2 and weight <70 kg | Unpublished: Effect of Polyphenols on Iron Absorption from Finger Millet | - |
| 24 | 2018 | 105 | 1 | 18-40 | 18.5-25 kg/m2 and weight <65 kg | Unpubished: An Iron Isotope Study in Humans to Evaluate the Iron Bioavailability of a Novel Iron Compound - Iron Fatty Acid Complex | - |
| 25 | 2020 | 140 | 1 | 18-45 | 18.5-25 kg/m2 and weight <70 kg | Prebiotic Galacto-Oligosaccharides and Fructo-Oligosaccharides, but Not Acacia Gum, Increase Iron Absorption from a Single High-Dose Ferrous Fumarate Supplement in Iron-Depleted Women | 17 |
| 26 | 2020 | 86 | 1 | 18-40 | 18.5-25 kg/m2 and weight <70 kg | Unpublished: Iron absorption from sodium iron chlorophyllin is lower than from ferrous sulfate but is less affected by ascorbic acid and polyphenols: stable iron isotope studies in young women | - |

^1^ Study type: 1=absorption study screening participants; 2=intervention study screening participants; 3=national, cross-sectional study

^2^ These were the criteria for inclusion in the study the screening was done for. Thus participants outside these criteria were still included in the present analysis if they presented for screening.

**Supplementary Table 2** Spearman correlation matrix of ferritin, Hb, BMI, age and CRP in participants without inflammation (based on a CRP < 5 mg/l) (exact n for each association shown in the table)

|  |  | **BMI** | **Age** | **Hb** | **CRP** |
| --- | --- | --- | --- | --- | --- |
| **Ferritin** | **r** | 0.66 | 0.48 | 0.283 | 0.028 |
|  | **p** | 0.001 | 0.022 | <0.001 | 0.161 |
|  | **n** | 2462 | 2324 | 2455 | 2469 |
| **BMI** | **r** |  | 0.077 | 0.023 | 0.183 |
|  | **p** |  | <0.001 | 0.254 | <0.001 |
|  | **n** |  | 2320 | 2451 | 2462 |
| **Age** | **r** |  |  | -0.044 | -0.025 |
|  | **p** |  |  | 0.034 | 0.234 |
|  | **n** |  |  | 2315 | 2324 |
| **Hb** | **r** |  |  |  | -0.025 |
|  | **p** |  |  |  | 0.223 |
|  | **n** |  |  |  | 2455 |

**Supplementary Table 3** Regression analyses including participants without inflammation (based on a CRP < 5 mg/l)

| **Model** | **Dependent** | **Independent** | **B** | **Beta** | **p** | **R^2^** |
| --- | --- | --- | --- | --- | --- | --- |
| 1 | Log ferritin | BMI | 0.010 | 0.067 | 0.002 | 0.011 |
|  |  | Age | 0.005 | 0.070 | <0.001 |  |
|  |  | Log CRP | 0.010 | 0.006 | 0.793 |  |
| 2 | Log ferritin | BMI | 0.010 | 0.068 | 0.001 | 0.011 |
|  |  | Age | 0.005 | 0.070 | 0.001 |  |
| 3 | Hb | BMI | 0.008 | 0.019 | 0.342 | 0.145 |
|  |  | Age | -0.014 | -0.073 | <0.001 |  |
|  |  | Log CRP | -0.197 | -0.040 | 0.040 |  |
|  |  | Log Ferritin | 1.015 | 0.377 | <0.001 |  |
| 4 | Hb | Age | -0.014 | -0.071 | <0.001 | 0.144 |
|  |  | Log CRP | -0.182 | -0.037 | 0.053 |  |
|  |  | Log Ferritin | 1.017 | 0.378 | <0.001 |  |

BMI: body mass index; CRP: C-reactive protein, Hb: hemoglobin

**
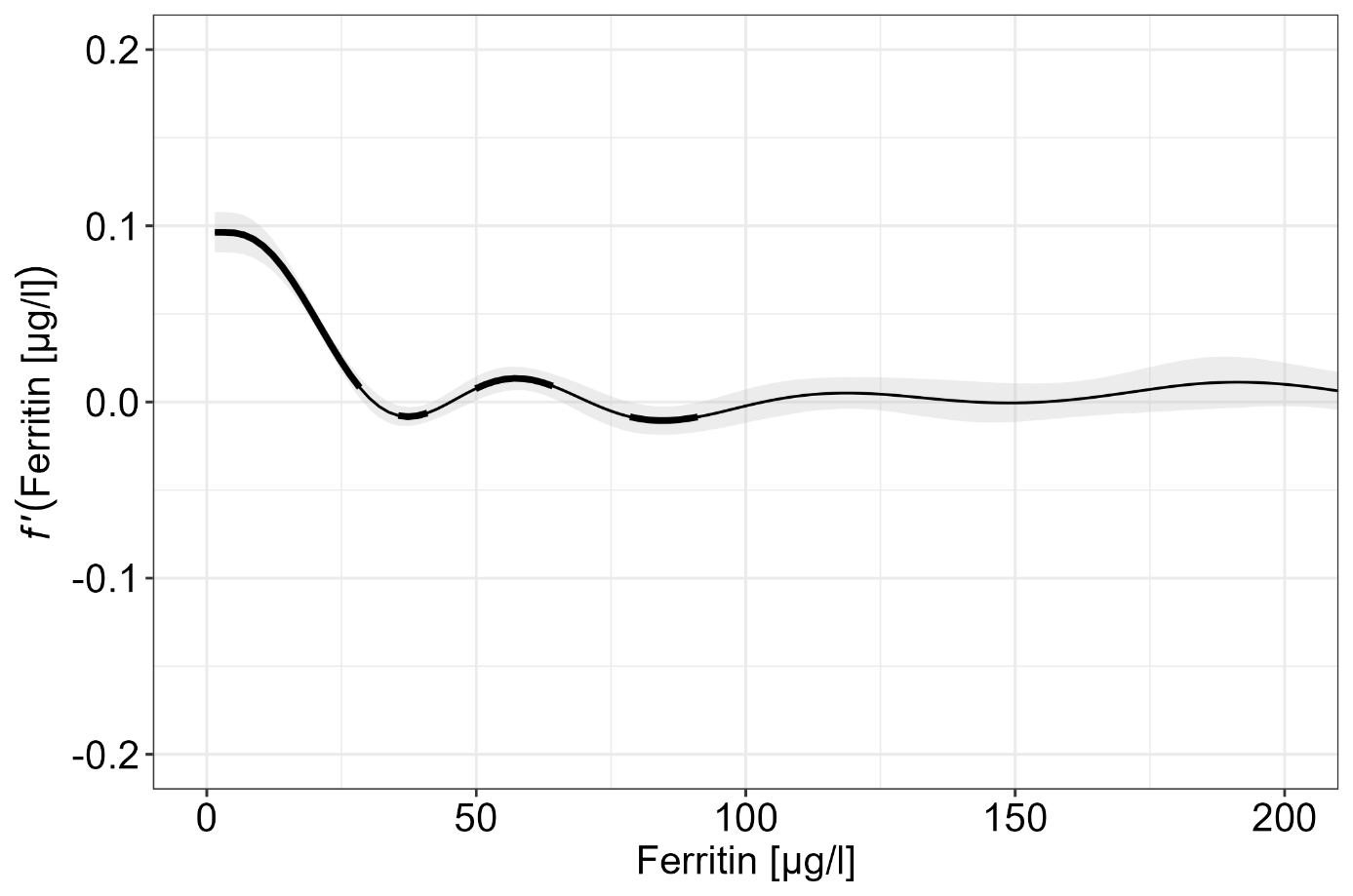
**

**Supplemental Figure 1** The first derivatives of the fitted GAMM shown in Figure 1 (ferritin versus hemoglobin) is shown for ferritin (n = 2453), excluding inflamed subjects (based on a CRP ≥ 5 mg/l). The line shows the estimate first derivative based on 10’000 posterior simulations and the shaded area the upper and lower 95% confidence intervals around the estimate. The thick line shows the sections where the first derivative departs significantly from zero, i.e. where the 95% confidence interval does not include zero. The derivative indicates that the model’s slope in Figure 1 is significantly positive from a ferritin value of 1.5 until 28.5 µg/l.

**References**

1. Speich C, Mitchikpe CES, Cercamondi CI, Zeder C, Brittenham GM, Moretti D, et al. Direct assessment of body iron balance in women with and without iron supplementation using a long-term isotope dilution method in Benin and Switzerland. Am J Clin Nutr. 2021;113(6):1657-69. doi: 10.1093/ajcn/nqaa433. PubMed PMID: WOS:000658172100032.
2. Anselmo AC, Xu X, Buerkli S, Zeng YY, Tang W, McHugh KJ, et al. A heat-stable microparticle platform for oral micronutrient delivery. Sci Transl Med. 2019;11(518). doi: ARTN eaaw3680
3. Scheuchzer P, Zimmerman MB, Zeder C, Sanchez-Ferrer A, Moretti D. Higher Extrusion Temperature Induces Greater Formation of Less Digestible Type V and Retrograded Starch in Iron-Fortified Rice Grains But Does Not Affect Iron Bioavailability: Stable Isotope Studies in Young Women. J Nutr. 2022;152(5):1220-7. doi: ARTN nxab435
4. Jeroense FMD, Zeder C, Zimmermann MB, Herter-Aeberli I. Acute Consumption of Prebiotic Galacto-Oligosaccharides Increases Iron Absorption from Ferrous Fumarate, but not from Ferrous Sulfate and Ferric Pyrophosphate: Stable Iron Isotope Studies in Iron-Depleted Young Women. J Nutr. 2020;150(9):2391-7. doi: 10.1093/jn/nxaa199. PubMed PMID: WOS:000568434200021.
5. Jeroense FMD, Michel L, Zeder C, Herter-Aeberli I, Zimmermann MB. Consumption of Galacto-Oligosaccharides Increases Iron Absorption from Ferrous Fumarate: A Stable Iron Isotope Study in Iron- Depleted Young Women. J Nutr. 2019;149(5):738-46. doi: 10.1093/jn/nxy327. PubMed PMID: WOS:000466858600007.
6. Andersson M, Theis W, Zimmermann MB, Foman JT, Jäkel M, Duchateau G, et al. Random serial sampling to evaluate efficacy of iron fortification: a randomized controlled trial of margarine fortification with ferric pyrophosphate or sodium iron edetate. Am J Clin Nutr. 2010;92(5):1094-104. doi: 10.3945/ajcn.2010.29523.
7. Herter-Aeberli I, Fischer MM, Egli IM, Zeder C, Zimmermann MB, Hurrell RF. Addition of Whole Wheat Flour During Injera Fermentation Degrades Phytic Acid and Triples Iron Absorption from Fortified Tef in Young Women. J Nutr. 2020. Epub 2020/08/18. doi: 10.1093/jn/nxaa211. PubMed PMID: 32805002.
8. Cepeda-Lopez AC, Melse-Boonstra A, Zimmermann MB, Herter-Aeberli I. In overweight and obese women, dietary iron absorption is reduced and the enhancement of iron absorption by ascorbic acid is one-half that in normal-weight women. Am J Clin Nutr. 2015;102(6):1389-97. doi: 10.3945/ajcn.114.099218. PubMed PMID: WOS:000365717300013.
9. Stoffel NU, Cercamondi CI, Brittenham G, Zeder C, Geurts-Moespot AJ, Swinkels DW, et al. Iron absorption from oral iron supplements given on consecutive versus alternate days and as single morning doses versus twice-daily split dosing in iron-depleted women: two open-label, randomised controlled trials. Lancet Haematol. 2017;4(11):E524-E33. doi: 10.1016/S2352-3026(17)30182-5. PubMed PMID: WOS:000414071900010.
10. Petry N, Egli I, Chassard C, Lacroix C, Hurrell R. Inulin modifies the bifidobacteria population, fecal lactate concentration, and fecal pH but does not influence iron absorption in women with low iron status. Am J Clin Nutr. 2012;96(2):325-31. doi: 10.3945/ajcn.112.035717. PubMed PMID: WOS:000306769700015.
11. Brittenham GM, Andersson M, Egli I, Foman JT, Zeder C, Westerman ME, et al. Circulating non-transferrin-bound iron after oral administration of supplemental and fortification doses of iron to healthy women: a randomized study. Am J Clin Nutr. 2014;100(3):813-20. doi: 10.3945/ajcn.113.081505. PubMed PMID: WOS:000340738800011.
12. Cercamondi CI, Icard-Verniere C, Egli IM, Vernay M, Hama F, Brouwer ID, et al. A Higher Proportion of Iron-Rich Leafy Vegetables in a Typical Burkinabe Maize Meal Does Not Increase the Amount of Iron Absorbed in Young Women. J Nutr. 2014;144(9):1394-400. doi: 10.3945/jn.114.194670. PubMed PMID: WOS:000340736300007.
13. Cercamondi CI, Duchateau GSMJE, Harika RK, van den Berg R, Murray P, Koppenol WP, et al. Sodium pyrophosphate enhances iron bioavailability from bouillon cubes fortified with ferric pyrophosphate. Brit J Nutr. 2016;116(3):496-503. doi: 10.1017/S0007114516002191. PubMed PMID: WOS:000380907000012.
14. Cercamondi CI, Egli IM, Zeder C, Hurrell RF. Sodium iron EDTA and ascorbic acid, but not polyphenol oxidase treatment, counteract the strong inhibitory effect of polyphenols from brown sorghum on the absorption of fortification iron in young women. Brit J Nutr. 2014;111(3):481-9. doi: 10.1017/S0007114513002705. PubMed PMID: WOS:000332451700012.
15. Andersson M, Hunziker S, Fingerhut R, Zimmermann MB, Herter-Aeberli I. Effectiveness of increased salt iodine concentration on iodine status: trend analysis of cross-sectional national studies in Switzerland. European journal of nutrition. 2019. Epub 2019/03/08. doi: 10.1007/s00394-019-01927-4. PubMed PMID: 30843107.
16. Husmann FMD, Stierli L, Bram DS, Zeder C, Kramer SD, Zimmermann MB, et al. Kinetics of iron absorption from ferrous fumarate with and without galacto-oligosaccharides determined from stable isotope appearance curves in women. Am J Clin Nutr. 2022;115(3):949-57. doi: 10.1093/ajcn/nqab361. PubMed PMID: WOS:000764264200035.
17. Giorgetti A, Husman FMD, Zeder C, Herter-Aeberli I, Zimmermann MB. Prebiotic Galacto-Oligosaccharides and Fructo-Oligosaccharides, but Not Acacia Gum, Increase Iron Absorption from a Single High-Dose Ferrous Fumarate Supplement in Iron-Depleted Women. J Nutr. 2022. doi: 10.1093/jn/nxac003. PubMed PMID: WOS:000756890100001.
